# Identification and characterisation of microRNAs and their target genes in phosphate-starved *Nicotiana benthamiana* by small RNA deep sequencing and 5’RACE analysis

**DOI:** 10.1186/s12864-018-5258-9

**Published:** 2018-12-17

**Authors:** Amanda Huen, Julia Bally, Penelope Smith

**Affiliations:** 1 School of Life and Environmental Sciences, The University of Sydney, Camperdown, NSW 2006 Australia; 2 Centre for Tropical Crops and Biocommodities, Queensland University of Technology, QLD, Brisbane, 4000 Australia; 3 Department of Animal, Plant and Soil Sciences, La Trobe University, Bundoora, VIC 3086 Australia

**Keywords:** Deep sequencing, Degradome, miRNA, *Nicotiana benthamiana*, Phosphate starvation, Small RNA, Transcript

## Abstract

**Background:**

Phosphorus is an important macronutrient that is severely lacking in soils. In plants, specific microRNAs (miRNAs) essential for nutrient management and the regulation of stress responses are responsible for the control of many phosphate starvation responses. Further understanding of conserved and species-specific microRNA species has potential implications for the development of crops tolerant to soils with low phosphate.

**Results:**

This study identified and characterised phosphate starvation-responsive miRNAs in the native Australian tobacco *Nicotiana benthamiana*. Small RNA libraries were constructed and sequenced from phosphate-starved plant leaves, stems and roots. Twenty-four conserved miRNA families and 36 species-specific miRNAs were identified. The majority of highly phosphate starvation-responsive miRNAs were highly conserved, comprising of members from the miR399, miR827, and miR2111 families. In addition, two miRNA-star species were identified to be phosphate starvation-responsive. A total of seven miRNA targets were confirmed using RLM-5’RACE to be cleaved by five miRNA families, including two confirmed cleavage targets for Nbe-miR399 species, one for Nbe-miR2111, and two for Nbe-miR398. A number of *N. benthamiana*-specific features for conserved miRNAs were identified, including species-specific miRNA targets predicted or confirmed for miR399, miR827, and miR398.

**Conclusions:**

Our results give an insight into the phosphate starvation-responsive miRNAs of *Nicotiana benthamiana*, and indicate that the phosphate starvation response pathways in *N. benthamiana* contain both highly conserved and species-specific components.

**Electronic supplementary material:**

The online version of this article (10.1186/s12864-018-5258-9) contains supplementary material, which is available to authorized users.

## Background

Phosphorus is the second-most important plant macronutrient after nitrogen, and a major limiting factor for agricultural production [1, 2]. Worldwide, natural soil phosphorus is low and declining, and artificial application of phosphate fertilisers is heavily relied upon, in order to maintain current agricultural production [3]. However, a large portion of the supplemented phosphate is inaccessible to the plant, and certain forms of phosphate fertiliser are ineffective in some soils [4]. Due to the non-renewable nature of phosphate, and the current inefficiencies in fertiliser usage, recent studies aim to determine avenues by which efficient plant uptake and usage of soil phosphate can be enhanced.

Small RNAs (sRNAs) are 20–24 nt long RNA species whose interactions with gene pathways are significant in regulating plant development and response pathways [5]. The miRNA biosynthesis pathway is conserved in eukaryotes, and different miRNAs are crucial for development, response and reproduction during the plant lifecycle. Plant miRNAs are generated from longer messenger RNA (mRNA precursors), known as primary miRNAs (pri-miRNAs), which are transcribed from defined promoter-containing loci within the plant genome and contain a stem-loop structure enzymatically processed into a duplex composed of the mature miRNA and a near-complementary passenger or miRNA-star (miRNA*) strand. The mature miRNA strand is separated from the miRNA* and loaded into an ARGONAUTE family member protein, which recognises transcripts near-complementary to the miRNA sequence and effects transcript cleavage or translational repression of the transcript, resulting in gene silencing [5, 6].

miRNA-mediated gene silencing regulates a diverse range of plant processes, and a number of works have focused on miRNAs associated with nutrient stresses such as phosphate starvation [7–11]. For example, miR399 is one of the most abundant and highly upregulated miRNAs during phosphate starvation [9]. It targets *PHOSPHATE2* (*PHO2*), which codes for an ubiquitin-conjugating E2 enzyme involved in the ubiquitination pathway for the degradation of phosphate transporters [12].

Australian soils, especially the arid interior, are some of the most phosphate-deprived in the world [13]. The native Australian tobacco *N. benthamiana,* a close relative of commercial tobacco (*Nicotiana tabacum*) and a common model for plant virus infection studies and transient gene expression assays, originates from a defence-deficient mutant population that has survived in the extreme habitat of Central Australia [14]. It is possible that *N. benthamiana* possesses unique miRNA regulation pathways that enable its survival within such a severe growing environment.

In this study, we characterised the sRNA profile of leaf, stem and root tissue of *N. benthamiana* under phosphate sufficiency (+P) and phosphate starvation (-P). Twenty-four conserved miRNA gene families and 36 species-specific miRNAs were identified from sRNA deep sequencing reads. We computationally predicted the phosphate starvation-responsive (PSR) miRNAs present in these samples and their corresponding miRNA targets. We experimentally confirmed the expression and phosphate starvation responsiveness of a number of miRNAs and their targets. The potential interactions and features of the miRNA profile in *N. benthamiana*, and their possible roles in the phosphate starvation response of *N. benthamiana* are discussed.

## Results

### Analysis of small RNA sequencing reads

Deep sequencing of sRNAs from *N. benthamiana* leaves, stems, and roots grown under phosphate-sufficient (+P) or phosphate starvation (-P) conditions yielded a total of 40,047,335 input reads (Table 1). The raw reads of the two libraries were uploaded to the National Center of Biotechnology Information (NCBI) Sequence Read Archives (SRA) database, and the following accession numbers were obtained: SRR5186271 (Stem +P), SRR5186272 (Stem -P), SRR5186273 (Root +P), SRR5186274 (Root -P), SRR5186275 (Leaf +P), SRR5186276 (Leaf -P). The adaptor sequences were trimmed from the sequencing reads, they were filtered for quality and structural RNAs (rRNAs, tRNAs) were removed. Remaining reads were matched to the *N. benthamiana* genome (v. 0.3) [15], yielding a total of 18,104,221 reads, or 45.2% of the total input reads (Table 1). The least redundant reads were found in stem tissue under +P (40.9% of total reads unique), closely followed by roots (39.5% unique reads), while leaf tissue under +P had the highest read redundancy (24.4% unique reads). Only filtered reads of 20–22 nt were considered for the identification and characterisation of candidate miRNAs.

**Table 1:** Summary of small RNA deep sequencing data in six libraries

	Leaf		Stem		Root		Total
	+P	-P	+P	-P	+P	-P	
Input reads	3,795,314	4,716,270	8,727,396	4,128,374	11,519,516	7,160,465	40,047,335 (a)
Input reads (non-redundant)	868,291	1,622,029	2,742,250	1,624,231	3,000,466	2,335,245	12,192,512
Filter for quality, length	2,603,939	3,690,013	5,213,478	3,394,598	6,219,796	4,222,059	25,343,883
Filter for non-structural	2,347,583	3,348,874	2,963,108	3,058,748	3,434,446	2,951,462	18,104,221 (b)
Filtered non-redundant reads	575,059	1,249,586	1,212,863	1,208,898	1,134,530	1,164,834	6,545,770
Non-redundant/ Total (b)/(a) (%)	24.5	37.3	40.9	39.5	33.0	39.5	
Total reads/Input reads (%)	45.2%						

### Conservation and phosphate starvation-responsiveness of miRNAs in *N. benthamiana*

**Fig. 1:**
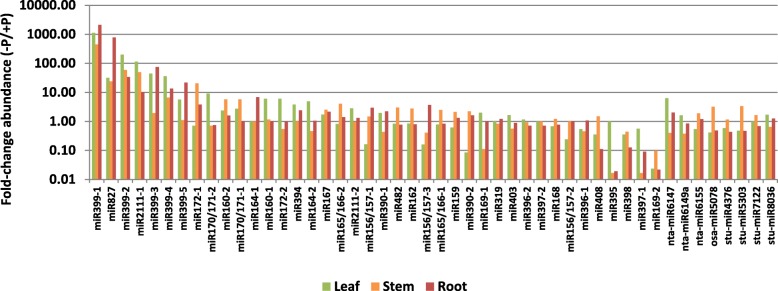
Phosphate response of miRNAs identified from sRNA deep sequencing reads in *N. benthamiana*. Fold-change of deep sequencing reads for miRNA family members. Numbers suffixed to miRNA names denote individuals of multiple miRNA family members. Poorly conserved miRNAs are denoted by the nearest miRNA homologue. Y-axis scale is in log_10_ of fold-change between phosphate starvation (-P) and phosphate sufficiency (+P) for normalised sequence counts per million (-P/+P)

**Fig. 2:**
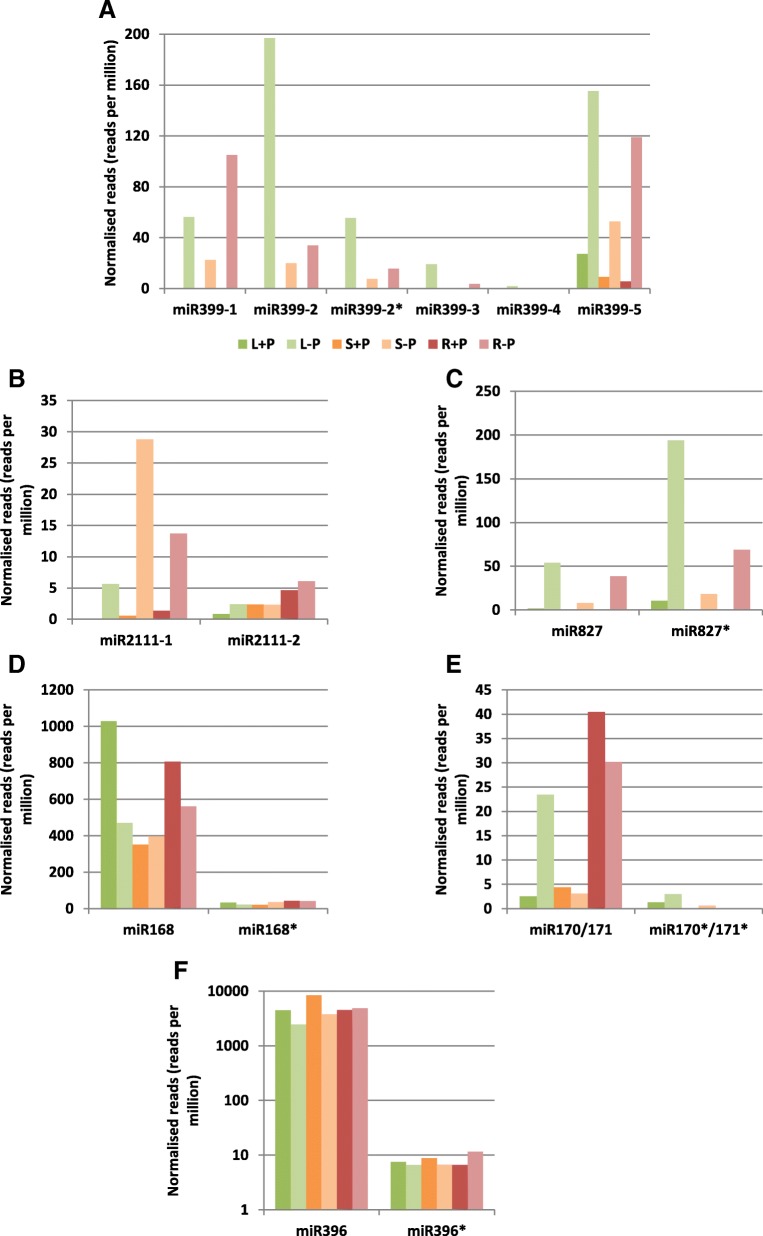
Small RNA deep sequencing read abundances of miRNAs and corresponding miRNAs*. **a**) miR399, **b**) miR2111, **c**) miR827, **d**) miR168, **e**) miR170/171, **f**) miR196. Eight week-old *N. benthamiana* plants were grown in phosphate-sufficient (+P) or phosphate-deficient (-P) quarter-strength liquid Hoagland medium for two weeks. Leaves (L), stems (S) and roots (R) were harvested

Twenty-four highly-conserved miRNA families were identified through sequence alignment to known plant miRNAs in the miRBASE database (v. 18), and with the miRProf program (Table 2). Several of the identified miRNAs belonged to phosphate responsive (PSR) miRNA families such as miR399, miR827, miR171, miR172, miR169, miR398, and miR408 [7, 9, 16], and their read abundances under -P were up to 1000-fold higher than +P (Fig. 1). For most of the conserved miRNAs, one or two family members with slight differences in their sequence were identified. Five miR399 members were identified (Table 2), and these are denoted miR399–1 to miR399–5 (Fig. 2a).

**Table 2:** Conservation of miRNA families in *N. benthamiana*

miRNA	Number of unique sequences in identified in *N. benthamiana* corresponding to previously identified conserved miRNAs	Conservation^a^ (no. of species)
miR156/157	3	all (7)
miR159	1	all (7)
miR160	2	all (7)
miR162	1	all (7)
miR164	2	all (7)
miR165/166	2	all (7)
miR167	1	all (7)
miR168	1	all (7)
miR169	2	all (7)
miR170/171	2	all (7)
miR172	2	all (7)
miR319	1	all (7)
miR390	2	all (7)
miR396	2	all (7)
miR397	2	all (7)
miR399	5	all (7)
miR394	1	N,A,P,O,S,Z (6)
miR395	1	N,A,M,P,O, Z (6)
miR398	1	N,A,M,P,O,Z (6)
miR408	1	N,A,M,P,O,Z (6)
miR482	1	N,M,P,S,Z (5)
miR827	1	N,A,P,O,Z (5)
miR2111	2	A,M,P (3)
miR403	1	A, P, S (3)
miR4376	1	S
miR473	1	P
miR5083	1	O
miR5303	1	O
miR6147	1	N
miR6149	1	N
miR6155	1	N
miR5078	1	O
miR7122	1	–

^a^N: *N. tabacum*, A: Arabidopsis, M: *M. truncatula*, P: *P. trichocarpa*, O: *O. sativa*, S: *S. lycopersicum*, Z: *Z. mays*

Of the miR399 members identified, miR399–1, miR399–2, and miR399–5 were the most abundant, with read numbers in excess of 20–200 reads per million for all three tissues under -P (Fig. 2a). miR399–5 was most abundant in roots and stems, while miR399–2 was most abundant in leaves. The abundance of both miR399–3 and miR399–4 also increased under -P but to a much lesser extent.

The mature Nbe-miR827 family member identified was homologous to miR827 in *N. tabacum* [17]. Its read abundance was significantly increased under -P in leaves and roots (Fig. 2c). Two miR2111 family members were identified and designated miR2111–1 and − 2 (Figs. 1 and 2b). miR2111–1 was elevated under -P, especially in stems (Fig. 2b), but miR2111–2 was not very responsive to -P, and remained at low levels in all tissues.

A number of poorly-conserved miRNAs were also identified. Matching of sRNA sequencing reads against the *N. benthamiana* draft genome using miRCAT identified 211 potential miRNAs as being potentially derived from a hairpin pri-miRNA, and 36 of these had detectable corresponding miRNA* reads in the dataset (Additional file 1: Table S1). A number of these miRNAs had enhanced expression during -P. For example, the homologues of miR5078 and miR5303 were increased over three-fold in the stem, and a miR6147 homologue was 6.3-fold more abundant in leaves grown in -P conditions than in those from +P plants (Fig. 1).

### miRNA-star species in *N. benthamiana*

A number of miRNA* species were identified, including miRNA* sequences for miR399–2 and miR827. miR399–2* reads were more abundant than miR399–2 under -P (22- to 31-fold greater, Fig. 2a). miR827* reads were also higher than miR827 in all tissues during -P (1.8-, 2.2- and 3.6-fold in roots, stems and leaves respectively), and 6.2-fold higher in leaves under +P (Fig. 2c). Other miRNA* reads were identified for miR168, miR170/171 and miR396; these were much lower in abundance than their corresponding miRNAs, being typically less than 10 reads per million and 10% of miRNA reads in each tissue (Fig. 2d–f).

### Validation of candidate phosphate starvation-responsive miRNAs in *N. benthamiana*

Selected miRNA species were chosen for further study according to their responsiveness to phosphate starvation in at least one tissue (leaves, stem, roots, Table 3), and the location of matching sequences in the genome in predicted pri-miRNA stem-loop structures (Additional file 2: Figure S1). A number of the selected miRNAs were from strongly conserved families; these were consistently increased in abundance under -P. miR399–1, miR399–2, miR399–2*, miR399–5, and miR827 had the highest relative change in abundance under -P (Fig. 2a,c). The miR482 homologue showed moderate change in shoot miRNA abundance but less change in roots (Table 3). miR482 was the most abundant miRNA of the 12 selected candidates, with in excess of 300–4700 reads per million, and a three-fold increase in read abundance in stem tissue under –P (Table 3).

**Table 3:** Small RNA deep sequencing read counts for miRNAs selected for further analysis

miRNA ID	Leaf			Stem			Root		
	Normalised weighted count (reads per million)								
	+P	-P	-P/+P	+P	-P	P/+P	+P	-P	-P/+P
miR01	768.88	340.11	0.44	26.66	97.43	3.65	9.61	4.07	0.42
miR6147	14.06	88.69	6.31	26.32	10.46	0.4	21.84	44.05	2.02
miR399–2	1	56.44	56.44	1	22.56	22.56	1	105.03	105.03
miR399–3	1	197.08	197.08	0.34	19.94	58.65	1	33.88	33.88
miR399–5	27.26	155.28	5.7	9.11	52.64	5.78	5.53	118.92	21.5
miR827	1.7	54.05	31.79	0.34	8.17	24.03	1	38.62	38.62
miR482	4769.16	3981.94	0.83	673.95	2013.9	2.99	425.69	323.57	0.76
miR827*	10.65	193.8	18.2	1	18.31	18.31	0.87	68.78	79.06
miR09	8.95	53.45	5.97	13.16	5.23	0.4	2.62	36.25	13.84
miR399–2*	1	55.54	55.54	0.34	7.52	22.12	1	15.59	15.59
miR11	51.12	200.07	3.91	215.65	311.89	1.45	62.02	169.75	2.74
miR12	6.82	62.11	9.11	4.39	31.06	7.08	13.1	38.62	2.95

Values in bold indicate -fold change> 2 for –P samples relative to +P samples. miR827* and miR399–2* are miRNA-star sequences

**Fig. 3:**
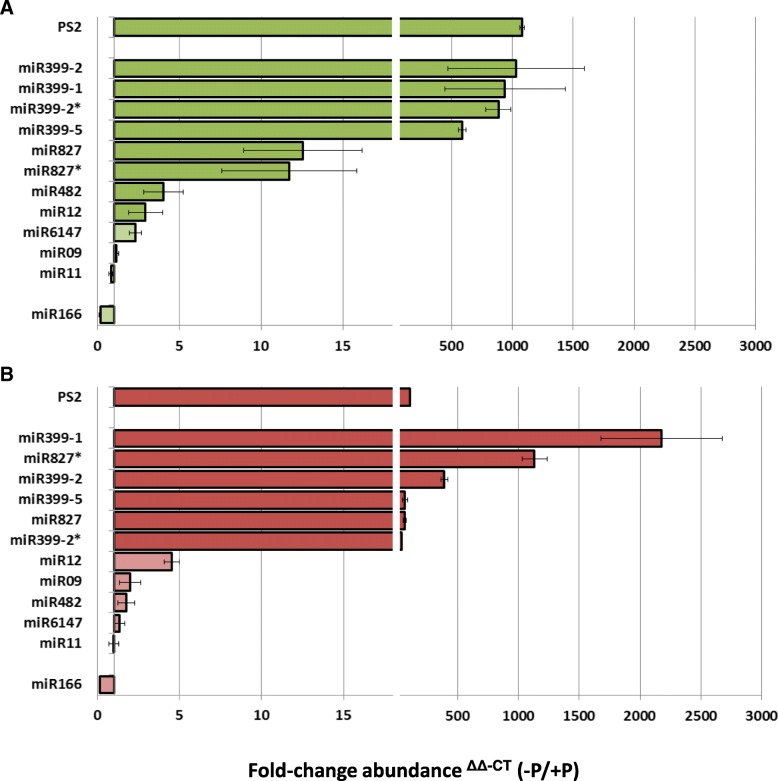
RT-qPCR measurement of candidate miRNA abundances in *N. benthamiana* **a**) shoots and **b**) roots under phosphate stress. *N. benthamiana* were grown as in Fig. 2. Shoots and roots were harvested. Average of target transcript abundance was calculated from three independent samples as measured by qPCR. miRNA abundance values were normalised to the Nbe-*UBC21* transcript and fold-change was calculated using the delta-delta –Ct method. Candidate miRNAs (miR02-miR12) are ordered according to fold-elevation in shoots and roots. *N. benthamiana PHOSPHATE STARVATION2* (*PS2*) is included as a phosphate starvation marker. Error bars indicate ±SE

In contrast to their phosphate starvation-responsiveness in the deep sequencing reads (Table 3), real-time-quantitative polymerase chain reaction (RT-qPCR) quantification of the poorly conserved miRNAs miR6147, miR09, and miR12 showed little or no responsiveness to phosphate starvation (Fig. 3).

**Fig. 4:**
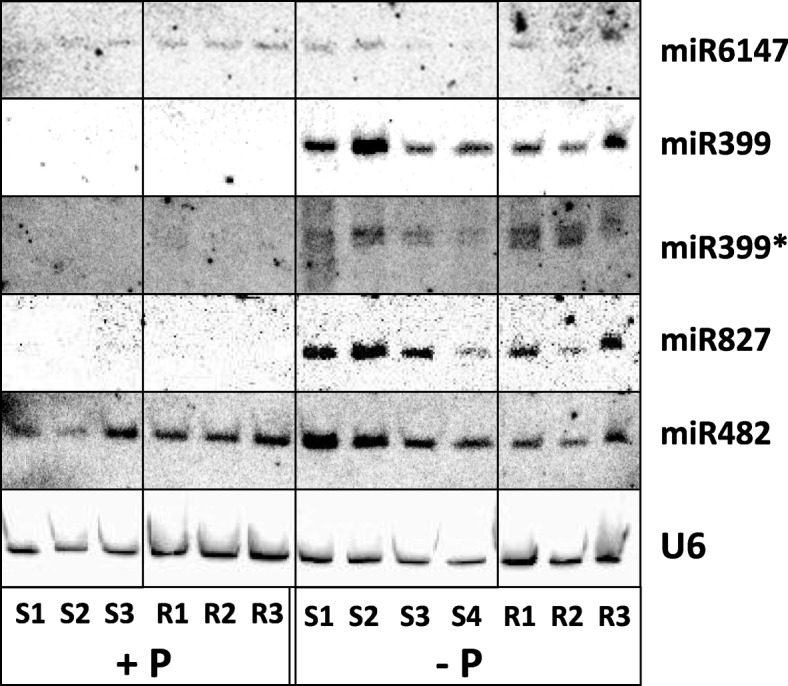
Northern blotting of phosphate starvation-responsive miRNAs. *N. benthamiana* were grown as in Fig. 2. 15μg total RNA from shoots (S) or roots (R) was blotted. 3–4 biological replicates were blotted per sample. *U6* was used as a loading standard

Northern blots were performed to confirm the phosphate starvation responsiveness of the candidate miRNAs. The miR399 band intensified under -P, as did miR399*, to a lesser extent (Fig. 4). None of the miR399 species were detected under +P. miR482 showed greater abundance in leaves under -P, and was also detectable under +P. miR827 increased in abundance especially in leaves under -P, but was not detectable under +P. A number of miRNA species could not be visualised successfully by Northern blot. miR827* could not be detected (data not shown), despite having a read number over 3.5-fold higher than miR827 (Fig. 2c). Additionally, miR2111*, miR2111, miR09, miR11, and miR12 were not identifiable by Northern blot (data not shown); these results may have been due to the comparatively lower abundances of these particular miRNA species (Table 3), or a higher rate of turnover.

### Promoter motif analysis in *MIRNA* genes

To determine potential *MIRNA* regulatory elements, stress response-associated *cis-*elements in *MIRNA* promoters for Arabidopsis and *N. benthamiana* were analysed using the PLACE online program [18]. The *PHOSPHATE STARVATION RESPONSE* (*PHR*) binding site (P1BS) is a conserved and well-characterised *cis*-element [19]. It consists of the sequence GNATATNC, and is bound by the MYB transcription factor PHR1 for the regulation of phosphate starvation-associated responses [20]. The site is commonly found in PSR gene promoters across different plant species [20]. One to four copies of the P1BS elements were present both in Arabidopsis and *N. benthamiana MIR399* and *MIR827* promoters, indicating the conserved influence of PHR1 on the expression of miR399 and miR827 in *N. benthamiana*. (Tables 4 and 5). Both Nbe-*MIR827* and Ath-*MIR827* promoters contained four copies of P1BS, and the *MIR1647* promoter contained two P1BS copies, suggesting that both loci are regulated by PHR1 in *N. benthamiana*. Ath-*MIR2111* promoters did not contain any P1BS copies (Tables 4 and 5), while one P1BS copy was predicted in the Nbe-*MIR2111–1* promoter (Table 5).

**Table 4:** Presence and number of motif copies in Arabidopsis *MIRNA* promoters

Motifs	miR399a	miR399b	miR399c	miR399d	miR399e	miR399f	miR827	miR2111a	miR2111b
TATA	YES	YES	ND^a^	YES	YES	YES	YES	YES	YES
P1BS	1	2	3	2	2	3	4	ND	ND
GATA-Box	3	> 10	> 10	4	4	> 10	6	> 10	> 10
W-box	> 10	4	7	ND	ND	5	9	9	4
LTRE	1	ND	ND	ND	ND	2	ND	3	1
DRE/C-repeat	ND	ND	ND	ND	ND	1	ND	1	ND

^a^ND: Not detected

**Table 5:** Presence and number of motif copies in *N. benthamiana MIRNA* promoters

Motifs	miR6147	miR399–1	miR399–2	miR399–3	miR399–4	miR399–5	miR827	miR2111–1
TATA	YES	YES	YES	YES	YES	YES	YES	YES
P1BS	2	1	3	3	2	3	4	1
GATA-Box	5	> 10	> 10	> 10	> 10	> 10	> 10	> 10
W-box	4	6	> 10	10	> 10	8	10	> 10
LTRE	ND^a^	2	ND	3	4	5	2	3
DRE/C-repeat	ND	1	1	1	ND	5	ND	1

^a^ND: Not detected

Other *cis-*elements associated with multiple abiotic stress-responses were surveyed. The DRE/C repeat is responsive to multiple abiotic stress responses including drought, temperature stress, and salt stress [21, 22]. Single copies of the dehydration-responsive element/C repeats (DRE/C repeats) were detected in all Nbe-*MIR399* promoters, with the exception of *MIR399–4*, which had no copies and *MIR399–5*, which had five copies (Table 5). In Arabidopsis, only the Ath-*MIR399f* promoter contained DRE/C repeats (Table 4). The Low Temperature Responsive Element (LTRE), which has been implicated in low-temperature, drought and light responses [23] was present in four out of the five Nbe-*MIR399* members surveyed (Table 5), and in Arabidopsis it was absent in all but two members (Table 4). One LTRE copy was predicted in Nbe-*MIR827*; this was absent in Arabidopsis.

### Prediction of miRNA targets

Target transcripts of identified miRNAs were computationally predicted using the psRNA Target Analysis program, with the *N. benthamiana* transcriptome (v.5.1) as the target transcript database (http://benthgenome.qut.edu.au/) [15]. All of the conserved miRNAs and miRNA*s analysed yielded at least one target (Table 6, Additional file 1: Table S2). The same three target transcripts were predicted for all five miR399 homologues (miR399–1-5): *PHO2*, a homologue to Ath-*PHT1;4*, and the transcript for the pattern formation protein EMBRYO DEFECTIVE30 (EMB30). For *PHO2*, four miR399 binding sites were identified, with binding affinities differing for each of the family members, and between the four binding sites (Table 6).

**Table 6:** Computationally predicted transcript targets of miR399 species identified from sRNA deep sequencing in *N. benthamiana*

Sequencing ID^a^ (miRNA name/number)	Nearest homologue	miRNA Sequence	miRNA Length	Expect^b^	Target Transcript, Target Accession^c^	Cleavage validation by 5’ RACE
B29_6_545_21_6_mir399 (miR399–1)	Ptc-miR399d	UGCCAAAGAAGAUUUGCCCCG	21	2	Inorganic phosphate transporter 1–4 (*PHT1;4*) (probable) Nbv5.1tr6377121	Yes
M00266_miR399 (miR399–3)	osa-miR399j	UGCCAAAGGAGAGUUGCCCUA		2		
B465_1_1169_21_2_mir399 (miR399–5)	Ath-miR399b	UGCCAAAGGAGAGUUGCCCUG		2		
B1376_1_8_21_2_mir399 (miR399–4)	osa-miR399i	UGCCAAAGGAGAGCUGCCCUG		3		
B947_1_816_21_1_NA (miR399–2)	Ptc-miR399f/g	UGCCAAAGGAGAAUUGUCCUG		3.5		
B947_1_816_21_1_NA (miR399–2)	Ptc-miR399f/g	UGCCAAAGGAGAAUUGUCCUG	21	2	Pattern formation protein: *EMB30* (probable) Nbv5.1tr6253408	No
M00266_miR399 (miR399–3)	osa-miR399j	UGCCAAAGGAGAGUUGCCCUA		3.5		
B465_1_1169_21_2_mir399 (miR399–5)	Ath-miR399b	UGCCAAAGGAGAGUUGCCCUG		3.5		
B29_6_545_21_6_mir399 (miR399–1)	Ptc-miR399d	UGCCAAAGAAGAUUUGCCCCG		4.5		
B1376_1_8_21_2_mir399 (miR399–4)	osa-miR399i	UGCCAAAGGAGAGCUGCCCUG		4.5		
B29_6_545_21_6_mir399 (miR399–1)	Ptc-miR399d	UGCCAAAGAAGAUUUGCCCCG	21	2.5	Probable *UBIQUITIN-CONJUGATING ENZYME E2 24* (probable) Nbv5.1tr6424601^d^	Yes (two sites)
				3		
				3		
				2		
B947_1_816_21_1_NA (miR399–2)	Ptc-miR399f/g	UGCCAAAGGAGAAUUGUCCUG	21	5		
				2		
				3.5		
				1.5		
M00266_miR399 (miR399–3)	osa-miR399j	UGCCAAAGGAGAGUUGCCCUA	21	4.5		
				3		
				3		
				1		
B1376_1_8_21_2_mir399 (miR399–4)	osa-miR399i	UGCCAAAGGAGAGCUGCCCUG	21	–		
				4		
				4		
				2		
B465_1_1169_21_2_mir399 (miR399–5)	Ath-miR399b	UGCCAAAGGAGAGUUGCCCUG	21	4.5		
				3		
				3		
				1		

^a^miRNA ID from *Nicotiana benthamiana* small RNA sequencing

^b^psRNA Target Analysis Expectation score ranges from 1 to 5, with a lower value indicating a better miRNA–target match

^c^Target Accession ID from *Nicotiana benthamiana* transcriptome v.5.1 (http://benthgenome.qut.edu.au/)

^d^All identified miR399 species in the table have three or four predicted binding sites in the Nbv5.1tr6424601 transcript

miR827 was predicted to target an SPX domain-containing protein, while miR827* matched a transcript for the homologue of ERF022, an ethylene-responsive transcription factor which modulates ethylene- and auxin-based growth responses for Arabidopsis somatic embryogenesis [24]. miR2111 matched the transcript for an F-box/kelch-repeat protein homologous to the Arabidopsis miR2111 target At3g27150, which is thought to participate in protein ubiquitination pathways [7, 9]. miR6147 matched a homologue encoding the late blight resistance protein R1B-16.

### miRNA target profiling by degradome sequencing

Degradome sequencing of miRNA targets was employed for high-throughput identification of potential miRNA targets. A total of 26,034,161 redundant raw reads were sequenced, with 1,686,008 (6.5%) of these reads mapping to the *N. benthamiana* transcriptome (Table 7). A total of 2133 reads matched to potential miRNA binding sites, with 1621 unique targets identified from these matches. A number of targets for conserved and species-specific miRNAs were identified from this list (Additional file 1: Table S3). However, few of the computationally predicted targets of *N. benthamiana* miRNAs were identified through degradome sequencing. Overall, degradome sequencing predicted targets for six conserved miRNA families and for 17 species-specific miRNAs (Additional file 1: Table S3). T-plots of selected transcripts are shown in Additional file 2: Figure S2.

**Table 7:** Summary of degradome sequencing data in three *N. benthamiana* tissue libraries

	Leaf	%	Stem	%	Root	%	Total	%
Raw reads	6,457,137		10,117,889		9,459,135		26,034,161	
Mapped reads	296,814	4.60	570,080	5.63	819,114	8.66	1,686,018	6.47
Reads matching miRNA cleavage	327		793		1013		2133	
		Non-redundant reads matching miRNA cleavage					1621	

The degradome reads (Additional file 1: Table S3) matched conserved miRNAs such as miR398 and miR2111, with conserved targets as well as species-specific targets computationally predicted from the *N. benthamiana* transcriptome (Table 6, Additional file 1: Table S2). miR399 was predicted to target transcripts for a DNA binding protein, a ribosomal protein, and a beta-glucanase protein, while the conserved *PHO2* target was not identified in the degradome reads. miR2111 was predicted to target transcripts related to ubiquitination (E3 ligase), and a bromodomain and plant homeodomain finger-containing protein which is likely to be involved in epigenetic regulation [25].

The abundance of miR398, a copper-induced miRNA [26], is decreased during phosphate starvation in *N. benthamiana* (Fig. 1). From degradome sequencing, the miR398 homologue was predicted to target three transcripts: a Cu/Zn superoxide dismutase (*CSD*), a homologue to an animal metallothionein family protein, and a blue copper binding protein (*BCBP*) (Additional file 1: Table S3).

**Fig. 5:**
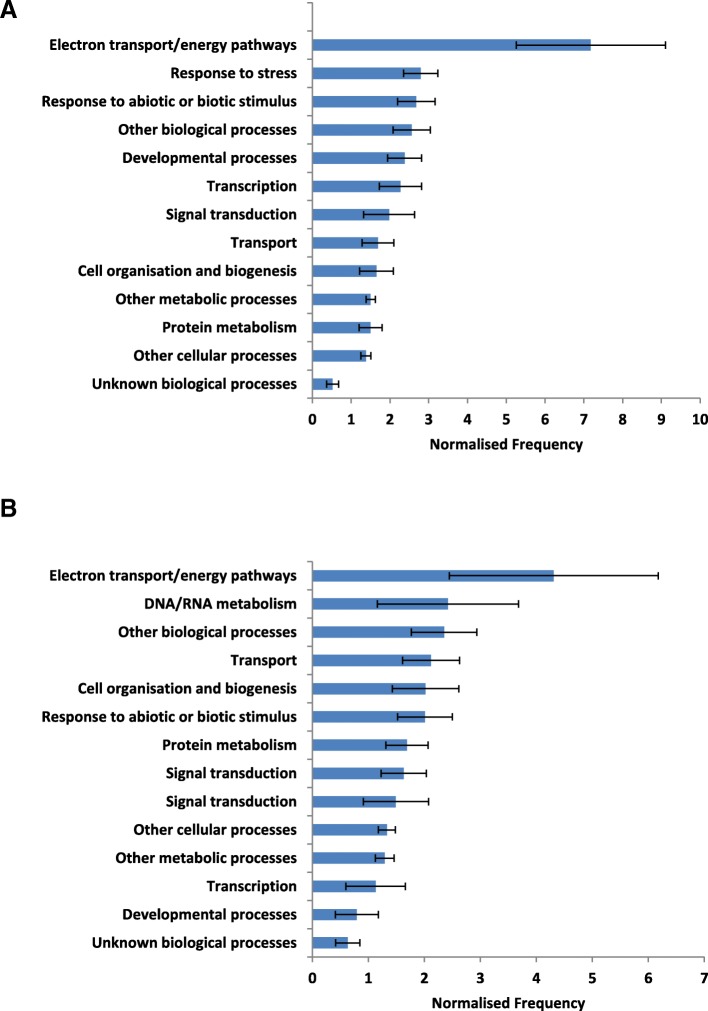
Gene ontology (GO) analysis and categorisation for Arabidopsis homologues of *N. benthamiana* miRNA targets predicted by degradome sequencing—novel and conserved targets. Ranked biological processes for transcripts identified by degradome sequencing as targets of **a**) conserved miRNAs and **b**) novel miRNAs

Gene ontology (GO) analysis and categorisation performed on the closest Arabidopsis homologues to the target transcripts predicted in the *N. benthamiana* miRNA degradome showed that the majority of conserved predicted targets were mainly associated with “Response to stresses” and “Response to abiotic or biotic stimulus” (Fig. 5a), while species-specific predicted targets were sorted mainly into “DNA/RNA metabolism” and “Other biological processes” (Fig. 5b).

### Validation of miRNA target cleavage using RLM-5’ RACE

**Fig. 6:**
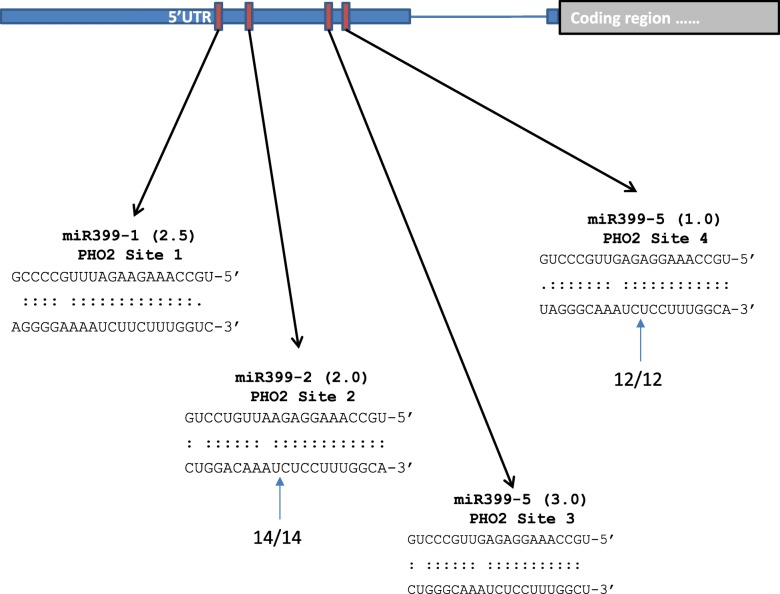
Computational prediction and experimental validation of *Nbe-*miR399 target *PHOSPHATE2* (*PHO2*). Top: Diagram of miR399 binding sites (red boxes) in the *PHO2* 5′ untranslated region (5’ UTR). Part of the *PHO2* coding region (grey) and an intron (blue line) is shown. Bottom: Sequence complementarity and cleavage in the *PHO2* binding sites. Sequences for the highest matching miR399 species is shown at the top and the corresponding binding site in *PHO2* is shown at the bottom. Bracketed number indicates the psRNA Target finder score (see Additional file 1: Table S2). Blue arrows and numbers indicate the proportion of 5’ RACE reads cleaved at the indicated sites

**Fig. 7:**
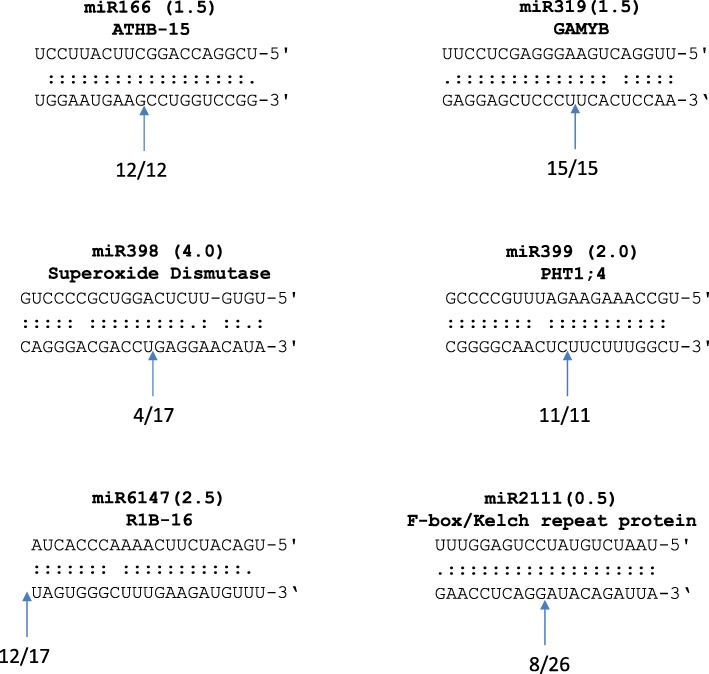
Sequence complementarity and cleavage in miRNA target transcripts as confirmed by 5’ RACE. *N. benthamiana* miRNAs and sequences are shown at the top and the corresponding binding site in the target transcripts is shown at the bottom. Bracketed number indicates the psRNA Target analysis Expect score (see Additional file 1: Table S2). Blue arrows and numbers indicate the proportion of RLM 5’ RACE reads cleaved at the indicated sites. Note that the cleavage site for *R1B-16* appeared to be at the beginning of the predicted miRNA binding site

**Fig. 8:**
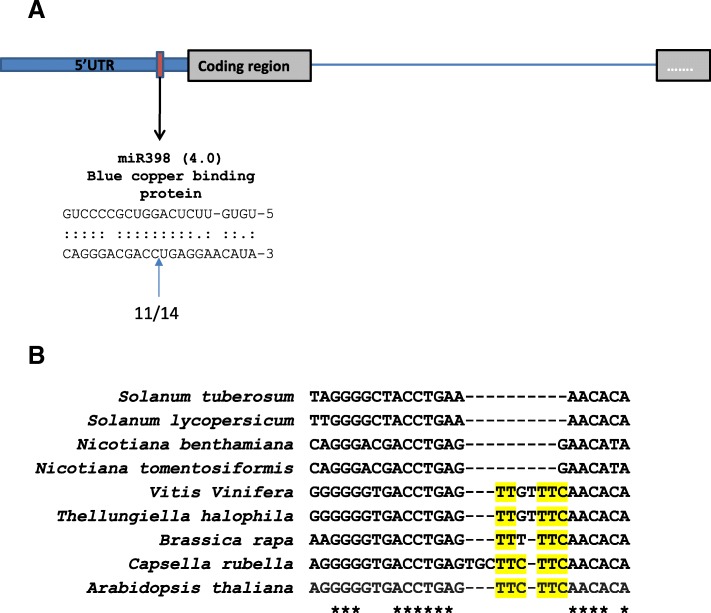
Cleavage of *N. benthamiana* blue copper binding protein (BCBP) transcript by miR398. **a**) Top: Diagram of *BCBP* showing position of miR398 binding site (red box) in the 5′ untranslated region (UTR, blue box). Part of the *BCBP* coding region (grey) and an intron (blue line) is shown. Bottom: Sequence complementarity and cleavage at the *BCBP* binding site. Bracketed number indicates the psRNA Target finder score (see Additional file 1: Table S3). Blue arrow and numbers indicate the proportion of 5’ RACE reads cleaved at the indicated site. **b**) Alignment of computationally identified miR398 binding sites in *BCBP* homologues. Highlighted regions in alignment indicate similarity between species at the six-nucleotide insert region found in Arabidopsis *BCBP*

Selected candidate *N. benthamiana* miRNA target transcripts were experimentally tested for miRNA cleavage using RLM-5’ RACE (henceforth referred to as 5’ RACE) (Table 8). The second and fourth binding sites out of the four sites predicted in *PHO2* were confirmed to be cleaved by miR399 (Fig. 6). *PHT1;4* was identified for the first time as a miR399 cleavage target in *N. benthamiana* (Table 8, Fig. 7). miR2111 was also confirmed to cleave the transcript for the miR2111 target, the F-box/kelch-repeat transcript (Fig. 7). Both *CSD* and *BCBP* were confirmed as targets of miR398 (Fig. 7, 8a).

**Table 8:** Target genes identified by 5’ RACE sequencing

miRNA ID^a^	Nearest homologue	Target	Clones matching	Region of cleavage site
B201_2_802950_21_4_mir166	Sly-miR166	Homeobox-leucine zipper protein ATHB-15 (probable)^b^	12/12	Coding
benth-B97_3_848_21_3_mir319	Sly-miR319	Transcription factor GAMYB (probable)^b^	15/15	Coding
B1569_1_1026_21_2_mir398	Nta-miR398	Superoxide dismutase [Cu-Zn] (similar to)^b^	4/17	5’UTR
		Blue copper binding protein (probable)^b^	11/14	5’UTR
B465_1_1169_21_2_mir399	Ath-miR399b	Probable ubiquitin-conjugating enzyme E2 24 (probable)^b^	14/14 (Site two) 12/12 (Site four)	5’ UTR
B29_6_545_21_6_mir399	Ptc-miR399d	Inorganic phosphate transporter 1–4 (probable)	11/11	Coding
B947_1_816_21_1_NA	Ptc-miR399f/g			
B246_2_947_21_2_NA	Nta-miR6147	Putative late blight resistance protein homolog R1B-16 (probable)	12/17 (Site two of two)^c^	Predicted as Coding
benth-B326_1_154_20_1_NA benth-B1461_1_95_20_1_NA	Ath-miR2111a-5p	F-box/kelch-repeat protein At3g27150 (probable)^b^	8/26	Coding

^a^miRNA ID from *Nicotiana benthamiana* small RNA sequencing reads

^b^Target previously confirmed in other species

^c^Cleavage point in all clones at the first nucleotide of miRNA binding site

Several tested transcripts could not be confirmed as cleaved miRNA targets, including *EMB30*, the SPX protein transcript, and *R1B-16*.

The transcript abundance of predicted miR399, miR2111, and miR6147 targets under +P and -P were quantified using RT-qPCR, but transcript levels were not significantly different between +P and -P (Additional file 2: Figure S3).

## Discussion

### Conserved and species-specific features of phosphate starvation-responsive miRNAs in *N. benthamiana*

Deep sequencing reads for miR399, miR827, and miR2111 were the most highly-elevated reads under –P. These miRNAs and their PSR responses are conserved across a number of plant species [27, 28], and their high conservation in *N. benthamiana* suggests that this species also possesses similarly conserved miRNA-regulated phosphate response pathways.

Conserved *N. benthamiana* miRNAs and targets showed evidence for differences in potential functions compared to other plant miRNA homologues. miR399 is the most abundant PSR miRNA expressed under phosphate starvation [9, 29], and is well-characterised as a regulator of shoot phosphate accumulation under phosphate starvation in all monocots and dicots studied so far [30]. However, functional specialisation of miR399 exists in certain species [11, 31]. The variation in miR399 read abundance in the leaves and roots of *N. benthamiana* suggested that the miR399 members are differentially expressed according to spatial or temporal factors in the plant. miR399–5 is relatively high abundant under +P conditions in *N. benthamiana*, suggesting a unique role under normal phosphate conditions. It may maintain a basal level of phosphate uptake during +P conditions, or prime the plant to respond to -P conditions more quickly. Interestingly, Nbe-miR399–5 is orthologous to Ath-miR399b/c, which has the highest abundance of the Ath-miR399 species shortly after the onset of phosphate starvation [32], and also exists at basal levels in Arabidopsis leaf veins when phosphate is present [33]. miR399 was also detected in the phloem of phosphate sufficient plants [34]. This indicates some homology of specialisation, and *N. benthamiana* may employ a similar PSR strategy for miR399.

Computationally-predicted targets for miR399 in *N. benthamiana* included both conserved and species-specific transcripts, suggesting that Nbe-miR399 functions in both conventional and specialised pathways. We confirmed that Nbe-miR399 targets a *PHT1;4* phosphate transporter transcript. miR399 is also computationally predicted to target various *PHT1* phosphate transporter transcripts in other plant species including Arabidopsis, peach, maize, soybean, and chickpea [7, 9, 35–38]. The predicted affinity of some Nbe-miR399 members for *PHT1;4* was demonstrated in this study (Table 6), and this may be used to modulate phosphate uptake depending on the requirements of the plant. This is similar in other plants. Only two out of five miR399 binding sites in *Hordeum vulgare* (barley) have been confirmed to be cleaved, while in comparison, four out of five sites are cleaved in Arabidopsis [32, 39, 40].

The cleavage of only some binding sites in *PHO2* by miR399, and their predicted differences in affinity, may allow for greater control of transcript stability through different permutations of miR399 binding. *PHO2* is thought to be translationally repressed, as well as cleaved, in Arabidopsis [9, 29], and miRNA binding sites in *PHO2* may be bound by miR399 for translational repression. miR172 and miR156 cause translational repression on their targets, as well as cleaving them [41], and the same is likely for miR399.

A non-conserved predicted Nbe-miR399 target, *EMB30*, is a GDP/GTP exchange factor that is involved in polarity formation and proper cell division in the developing plant embryo [42]. More recently, this protein has also been implicated in the control of lateral root development in mature plants [43]. *N. benthamiana* may regulate *EMB30* during phosphate starvation to enhance lateral root proliferation, to improve the range of the root system for scavenging phosphate. Although *EMB30* was not confirmed as a miR399 target by 5’ RACE, it is still possible that this transcript is regulated by miR399 through translational repression.

miR2111 was initially thought to be absent from Solanaceae [44, 45], and has only recently been identified in *N. tabacum* and tomato [17, 46]. The existence of miR2111 in *N. benthamiana,* as well as the conservation of its target across species, suggests that miR2111 has a conserved role during phosphate starvation. The read abundance of miR2111–1 and its target transcript were greater in the stems and roots than in the leaves, in agreement with the expression pattern of miR2111 and its target in Arabidopsis and *B. napus* [7, 9]. The presence of miR2111 in the stem tissue of *N. benthamiana* raised the possibility that it acts as a long-distance miRNA, like miR399 [32]; however miR2111 does not appear to be mobile in Arabidopsis [47]. The abundance of the F-box transcript in this study did not decrease during -P in *N. benthamiana* (Additional file 2: Figure S3); this was also the case for Arabidopsis, where miR2111 and the F-box transcript abundance both increased under phosphate starvation [7]. The F-box transcript may be silenced in a tissue-specific manner such that its abundance increases only in specific tissues during phosphate starvation, or it may be silenced through translational repression as well as transcript cleavage [7].

miR827 is highly-conserved in both monocots and dicots [28]. However, its target transcript is not as well-conserved as those for miR399 and miR2111. The SPX-domain protein transcript predicted to be targeted by Nbe-miR827 was not homologous to the Arabidopsis miR827 targets *NITROGEN LIMITATION ADAPTATION* (*NLA*), which encodes a SPX domain-containing E3 ligase [48]. This suggests that the Nbe-miR827 target may have a different role compared to the Arabidopsis target. In rice, miR827 targets transcripts for two SPX domain-containing membrane-localised protein transporters, rather than ubiquitin pathway-related targets (i.e. Ath-*NLA*) [49, 50]. These differences may indicate that the role of miR827 varies according to the requirements of different plant species.

We confirmed *BCBP* cleavage by miR398 in *N. benthamiana* (Fig. 8a, Table 8). In Arabidopsis and closely related species, *BCBP* is cleaved by miR398 through an unconventional base-pairing pattern with the binding site forming a 6-nt bulge when base-paired with miR398 [51]. This bulge does not occur in *N. benthamiana*, other *Nicotiana* species, or members of Solanaceae (Fig. 8b). The presence or absence of this feature in *BCBP* does not prevent miR398-mediated silencing of *BCBP* in Arabidopsis [51]. *CSD* was identified as a conserved target for miR398 (Table 8) [27, 52], while the prediction of a transcript for a metallothionein homologue as a target is novel, as no related miR398 target transcripts have been identified to date.

### Transcriptional control of phosphate starvation-responsive *MIRNA* genes

Nbe-*MIRNA* promoters contained more copies of general stress-response *cis-*elements surveyed than did Ath-*MIRNA* promoters, and these elements may have an effect on *MIRNA* expression in response to phosphate starvation. While most of the analysed *MIRNA* promoters contained the phosphate starvation response-specific P1BS *cis*-element, certain general stress response elements such as the DRE/C-repeat and LTRE motifs were also more highly represented. This suggests that *Nbe-MIR399*, -*MIR827*, and -*MIR2111* loci are under additional regulation that may enhance their PSR functions. As these elements are also featured in various stress-responsive genes for different stress conditions, they may allow these miRNA members in *N. benthamiana* to be induced under other stress conditions. For example, it is known that miR399, miR827, and miR2111 can be induced under drought and salinity stress in Arabidopsis, *Medicago* [53, 54], and *N. tabacum* [55], and that Osa-miR399 is also induced under various nutrient stresses [53].

### Presence and phosphate response of miRNA-star sequences

Certain miRNA*s persist alongside their corresponding miRNAs, in some cases at levels similar to, or exceeding the abundance of the miRNA [56, 57]. miR399* and miR827* were identified as PSR species in *N. benthamiana* (Fig. 2a, c). The abundance of both miRNA*s was elevated above their respective miRNAs in all tissues surveyed, especially in shoots. This suggests that they have a functional role in phosphate starvation alongside their respective miRNAs. In contrast, the expression of other identified miRNA*s (for miR168, miR170/171, miR396) was only a small fraction of total miRNA abundances in all tissues (Fig. 2d–f), making it unlikely that they have any significant function.

The detection of miR827* in *N. benthamiana* and not in Arabidopsis shows that miRNA* degradation is not necessarily sequence-dependent, and suggests that miR827* has a species-specific function in *N. benthamiana*. The persistence of miR827* at high levels has not been documented to date and may be specific to *N. benthamiana*; Ath-miR827* was not detected in phosphate-starved Arabidopsis [9] and it is thought to be quickly degraded. miR827* was predicted to target the transcript for a homologue to an Arabidopsis ethylene-responsive transcription factor (ERF022) that is involved in somatic embryogenesis [24]. The ERF022 homologue may have been adapted for phosphate response functions in *N. benthamiana*; however its status as a miR827* target has not been experimentally confirmed.

Unlike miR827*, miR399* persists in the phloem of phosphate-starved Arabidopsis [7, 9], and *B. napus* [9]. This suggests that miR399* is a functional, phloem-mobile species during phosphate starvation. miR399* is predicted to target a clathrin heavy chain protein in Arabidopsis; however this has not been experimentally confirmed [9]. Other miRNA*s are functional regulators of transcript expression in other plant species; miR169* and miR399* in *Medicago* regulate target transcripts distinct from the corresponding miRNAs [58], while in tomato, a number of conserved miRNA*s have an affinity to sequences in Tomato leaf curl virus, and are implicated in pathogen defence mechanisms [59]. Further work is thus needed to verify the potential roles of miRNA*s identified in this study.

## Conclusions

*N. benthamiana* contains phosphate starvation-responsive miRNAs that are highly conserved with other plant species. Twenty-four conserved miRNA families were identified in *N. benthamiana*, including the PSR miRNAs miR399, miR827, and miR2111. In addition, 36 species-specific miRNAs were identified. Evidence of different regulatory *cis-*elements in *MIR399*, *MIR827*, and *MIR2111* promoters suggests that these conserved *N. benthamiana* miRNAs have diverged from the typical phosphate starvation pathways seen in Arabidopsis and other species. The phosphate response and persistence of miR399* and miR827* in sequencing reads of *N. benthamiana* also suggests a role for these miRNA*s under phosphate starvation. *PHT1;4*, as well as *PHO2* were targeted by miR399, and *EMB30* was computationally identified as a potential miR399 target. The computationally predicted target for miR827, an SPX domain containing protein, was not homologous to the target for miR827 in other plant species. *BCBP* in *N. benthamiana* was targeted through conventional base-pairing with miR398 in *N. benthamiana*, unlike the Arabidopsis miR398/*BCBP* pairing. The unique features of the PSR miRNA pathways in *N. benthamiana* may explain the resistance of this species to phosphate starvation, and further study of the conserved and species-specific miRNAs identified in this work will help to uncover more details on PSR miRNA roles in *N. benthamiana*.

## Methods

### Plant growth and RNA extraction

Sterilised *Nicotiana benthamiana* seeds were germinated in half-strength Murashige and Skoog (MS) 0.8% (w/v) agar medium at 21 °C under a 16-h light/8-h dark photoperiod in a climate-controlled growth room. Eight-week old plants were transferred to complete quarter-strength Hoagland’s medium containing 0.5 mM KH_2_PO_4_. After two weeks, plants were transferred to media of the same composition (+P), or 0.62 mM K_2_SO_4_ in place of KH_2_PO_4_ (-P). Plant tissues were harvested after two weeks and stored at − 80 °C for RNA isolation. For plants grown on agar medium, seven day-old seedlings were transferred to half-strength Hoagland’s medium with 1% (w/v) sucrose containing 1 mM KH_2_PO_4_, or half-strength Hoagland’s medium with 1% (w/v) sucrose and 1.24 mM K_2_SO_4_ in place of KH_2_PO_4_. Plant tissues were harvested after eight days of treatment. Total RNA was extracted from *N. benthamiana* tissue using TRIzol (Life Technologies) according to the manufacturer’s instructions.

### Identification of small RNAs through deep sequencing

Small RNA libraries were prepared from total RNA for deep sequencing on the Illumina HiSeq-2000 platform according to manufacturer’s instructions. Library construction and deep sequencing was done by the Australian Genome Research Facility (AGRF, Parkville, VIC). Six libraries for leaf, stem and root under +P or –P treatments were pooled for sequencing using the Illumina HiSeq-2000 platform. Reads were adaptor-trimmed, pre-processed, and analysed using the University of East Anglia (UEA) sRNA Workbench [60]. Only trimmed reads 16–25 nt in length were considered for analysis. Conserved miRNAs were identified using by submitting the pre-processed sRNA reads to the miRProf program (UEA sRNA toolkit, Plant version). The sRNA reads were matched to known miRNAs listed in miRBase Database (v. 18), as well as tomato miRNAs from the Tomato Functional Genomics Database [61].

MicroRNA loci, primary structures and miRNA*s were predicted using the miRCAT program (UEA sRNA toolkit, Plant version). Predictions were made against the draft assembly of the *N. benthamiana* genome (v. 0.2) [15]. Targets for key identified miRNAs were computationally predicted using the psRNATarget program [62]. All miRNAs in this paper are referred to by their nearest homologue, and non-conserved miRNAs are referred to by their ID number as denoted in Additional file 1: Table S2.

### Quantitative PCR for small RNAs and targets

Total RNA was isolated from shoots and roots of *N. benthamiana* seedlings. Stem-loop cDNAs for specific miRNAs were synthesised from total RNA using the method according to Varkonyi-gasic and Hellens [63] with 100 ng total RNA and 2.5 U/μL SuperScript III reverse transcriptase (Life Technologies). Stem-loop RT-qPCR was performed using Brilliant II SYBR® Green (Agilent) according to the manufacturers’ directions. Poly(A)dT_23_ cDNAs were synthesized using 1 μg total RNA and SuperScript III according to the manufacturer’s directions. RT-qPCR was performed as before. Nbe-*UBC21* was used as a reference gene in both cases.

### Northern blotting of small RNAs

Fifteen micrograms of RNA was loaded into each lane of a 17% (w/v) polyacrylamide gel and resolved at 200 V for four hours. RNA was transferred electrophoretically to Hybond N+ membrane (GE Healthcare) by applying 90 V for 45 min. Membranes were crosslinked using a UV crosslinker (120 mJ cm^− 2^). DNA oligonucleotides complementary to the miRNAs of interest were end-labelled with ^32^P-dCTP (3000 Ci mmol^− 1^) using terminal DNA transferase (New England Biolabs) and hybridised to the membrane in PerfectHyb™ hybridisation buffer (Sigma) for 16 h at 42 °C. Membranes were washed three times for 20 min each in wash buffer (0.3 M NaCl, 0.15 M sodium citrate and 0.2% SDS) at 50 °C, and exposed to a K-screen for 1–3 days before viewing with a phosphorimager (Typhoon™ FLA 9000, GE Healthcare Life Sciences).

### Degradome sequencing and analysis

Degradome RNA libraries were prepared from total *N. benthamiana* RNA for sequencing on the Illumina HiSeq 2000 platform, as described in Jiang et al. [64] (LC Sciences, Houston, TX). Purified cDNA libraries were created from phosphate-starved *N. benthamiana* leaves, stems and roots, and sequenced on the Illumina HiSeq 2000 platform (LC Sciences, Houston, TX). The resulting 51 nt reads were adaptor-trimmed and filtered. Reads were aligned to a *N. benthamiana* Unigene dataset [15]. Potential miRNA cleavage sites were identified using the CleaveLand pipeline (v. 4.3) [65]. GO analysis was performed using the Classification Superviewer tool on the Bio-Analytic Resource website (v. 14–05, University of Toronto).

## Additional files

Additional file 1: **Table S1.** Microsoft Excel Worksheet (.xlsx). sRNA deep sequencing read matches to predicted pri-miRNA hairpins. **Table S2.** Computationally predicted miRNA targets in *N. benthamiana*. **Table S3.** Top targets of *N. benthamiana* miRNAs predicted through degradome sequencing. (XLSX 74 kb)

Additional file 2: **Figure S1.** Microsoft PowerPoint Presentation (.pptx). Predicted structures for *N. benthamiana* pri-miRNAs identified from sRNA deep sequencing. **Figure S2.**. Degradome T-plots for potential miRNA targets. **Figure S3.** Transcript abundance of targets tested by RLM 5’ RACE for cleavage by phosphate starvation-responsive miRNAs. (PDF 980 kb)

## Abbreviations

+P: Phosphate sufficiency
BCBP: Blue copper binding protein
CSD: Cu/Zn superoxide dismutase
DRE/C repeat: Dehydration-responsive element/C repeat
EMB30: EMBRYO DEFECTIVE30
LTRE: Low temperature responsive element
miRNA: MicroRNA
miRNA*: MicroRNA-star
mRNA: Messenger RNA
NLA: NITROGEN LIMITATION ADAPTATION
-P: Phosphate starvation
P1BS: PHOSPHATE STARVATION RESPONSE1 binding site
PHO2: PHOSPHATE2
PHR1: PHOSPHATE STARVATION RESPONSE1
pri-miRNA: Primary microRNA
PSR: Phosphate starvation-responsive
RLM-5’ RACE: RNA ligase-mediated 5’rapid amplification of complementary DNA ends
RT-qPCR: Real-time-quantitative polymerase chain reaction
SRA: Sequence Read Archive
sRNA: Small RNA
