# Supplementary material for: Identification and characterisation of microRNAs and their target genes in phosphate-starved Nicotiana benthamiana by small RNA deep sequencing and 5’RACE analysis
Source: BMC Genomics. 2018 Dec 17;19:940. doi: 10.1186/s12864-018-5258-9 (PMC6296076; doi:10.1186/s12864-018-5258-9)
Supplement: Supplementary file 2 — Figure S1. Microsoft PowerPoint Presentation (.pptx). Predicted structures for N. benthamiana pri-miRNAs identified from sRNA deep sequencing. Figure S2.. Degradome T-plots for potential miRNA targets. Figure S3. Transcript abundance of targets tested by RLM 5’ RACE for cleavage by phosphate starvation-responsive miRNAs. (PDF 980 kb) [file 12864_2018_5258_MOESM2_ESM.pdf]

Additional File 2

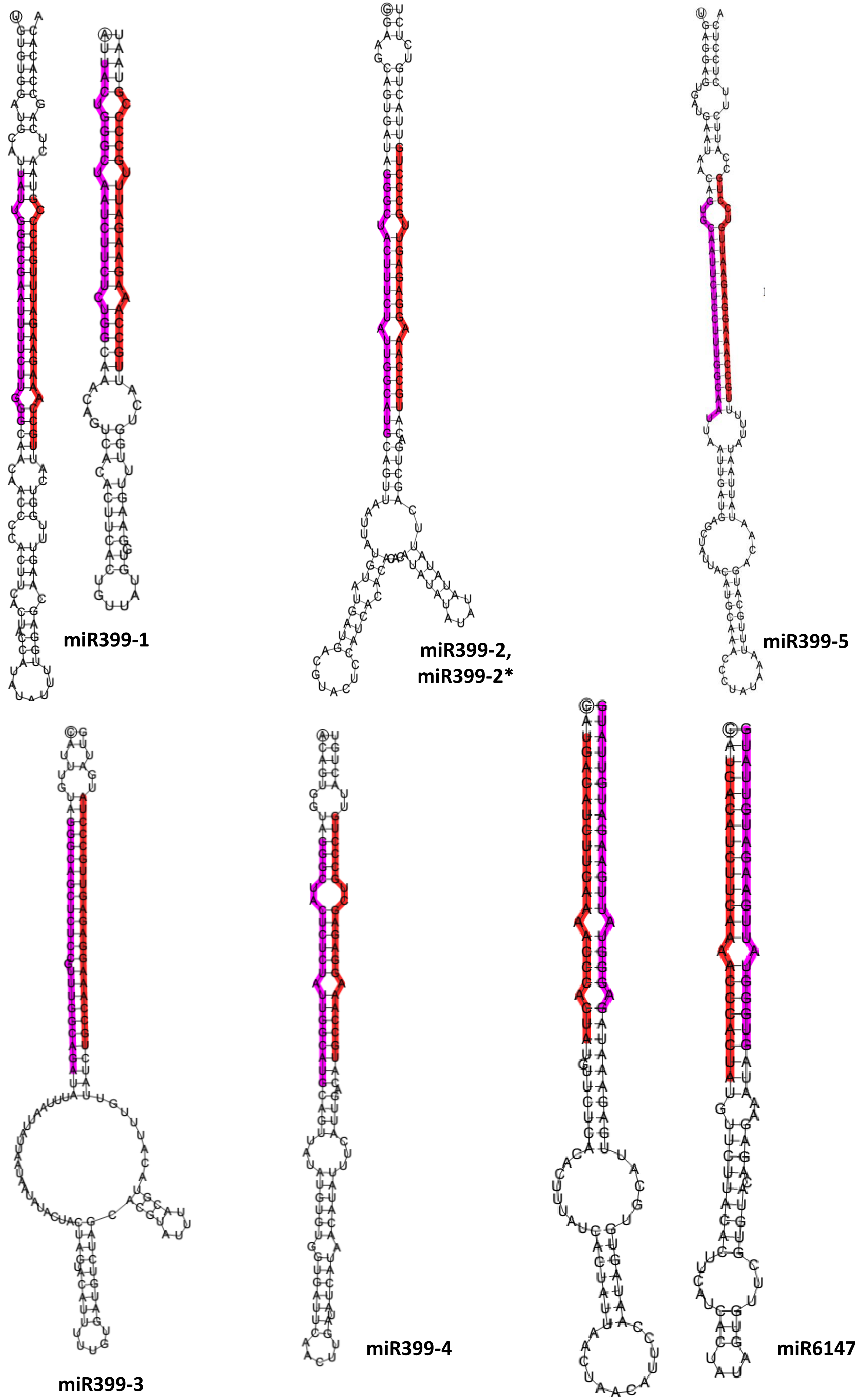
miR399-1
miR399-2,
miR399-2*
miR399-5
miR399-3
miR399-4
miR6147

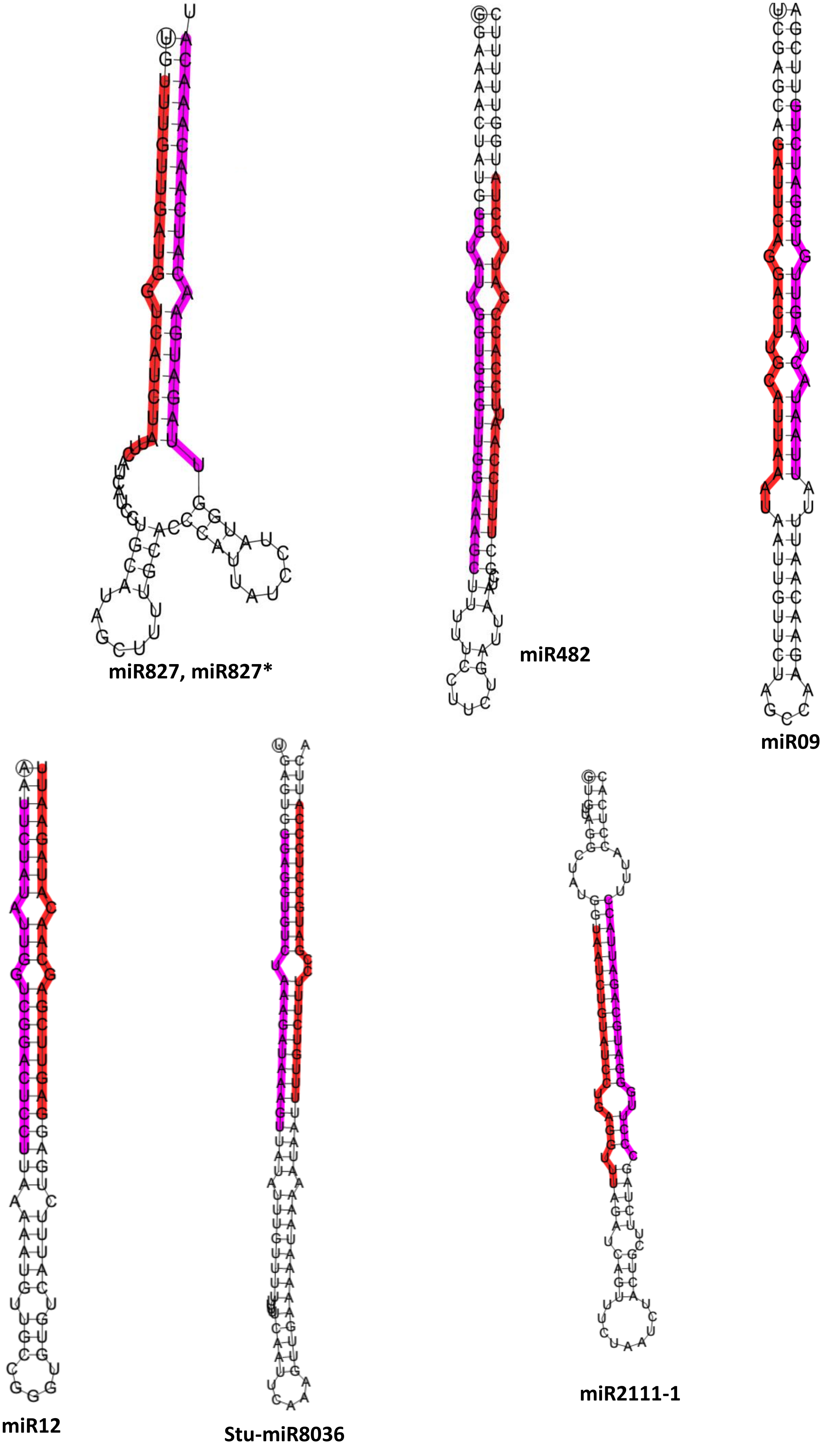
miR827, miR827*
miR482
miR09
miR12
Stu-miR8036
miR2111-1

Figure S1: Predicted structures for *N. benthamiana* pri-miRNAs identified from sRNA deep sequencing. Pri-miRNA structures were generated using the UEA RNAfold tool with default parameter settings. Red-highlighted nucleotides indicate the mature miRNA sequence while purple-highlighted nucleotides indicate the miRNA* sequence.

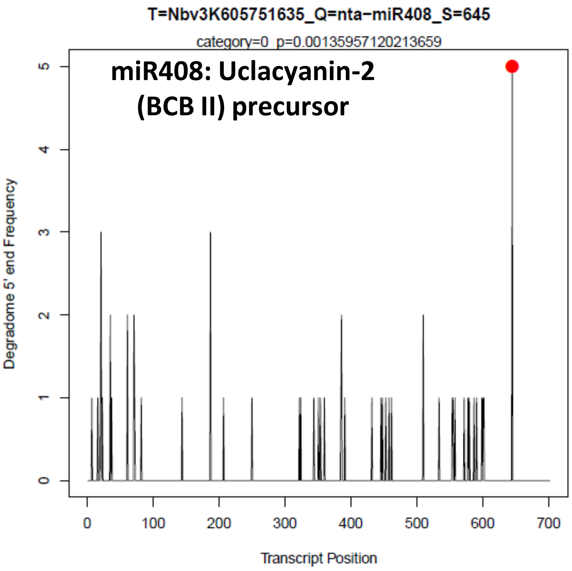
T=Nbv3K605751635_Q=nta−miR408_S=645
category=0 p=0.00135957120213659
miR408: Uclacyanin-2
(BCB II) precursor
Degradome 5' end Frequency
Transcript Position

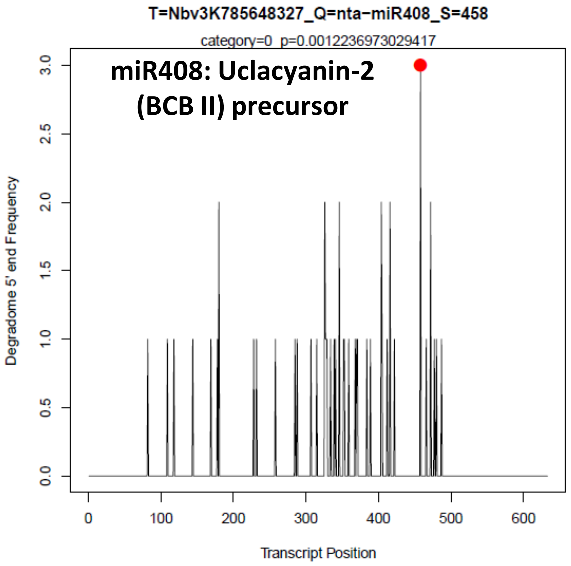
T=Nbv3K785648327_Q=nta−miR408_S=458
category=0 p=0.0012236973029417
miR408: Uclacyanin-2
(BCB II) precursor
Degradome 5' end Frequency
Transcript Position

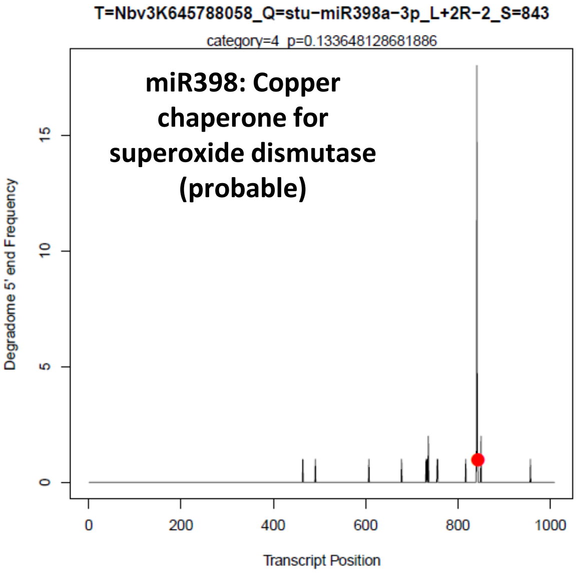
T=Nbv3K645788058_Q=stu−miR398a−3p_L+2R−2_S=843
category=4 p=0.133648128681886
miR398: Copper
chaperone for
superoxide dismutase
(probable)
Degradome 5' end Frequency
Transcript Position

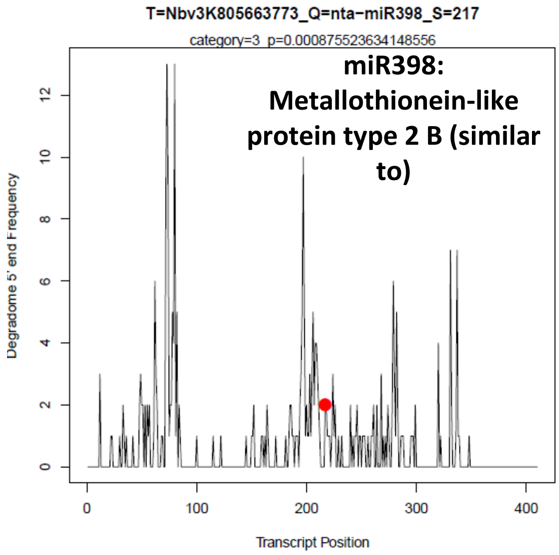
T=Nbv3K805663773_Q=nta−miR398_S=217
category=3 p=0.000875523634148556
miR398:
Metallothionein-like
protein type 2 B (similar
to)
Degradome 5' end Frequency
Transcript Position

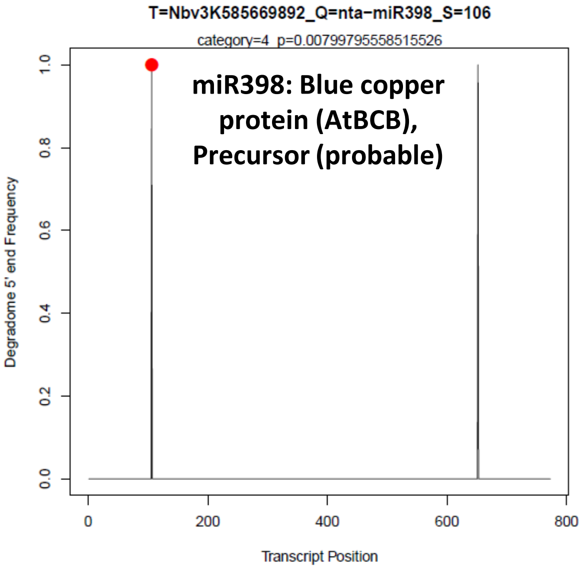
T=Nbv3K585669892_Q=nta−miR398_S=106
category=4 p=0.00799795558515526
miR398: Blue copper
protein (AtBCB),
Precursor (probable)
Degradome 5' end Frequency
Transcript Position

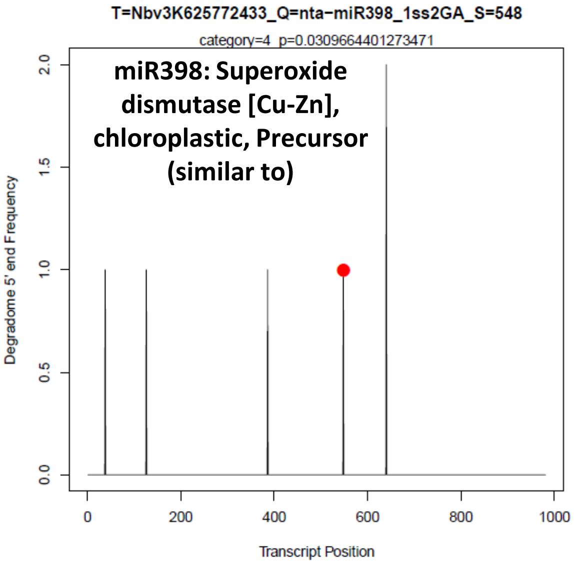
T=Nbv3K625772433_Q=nta−miR398_1ss2GA_S=548
category=4 p=0.0309664401273471
miR398: Superoxide
dismutase [Cu-Zn],
chloroplastic, Precursor
(similar to)
Degradome 5' end Frequency
Transcript Position

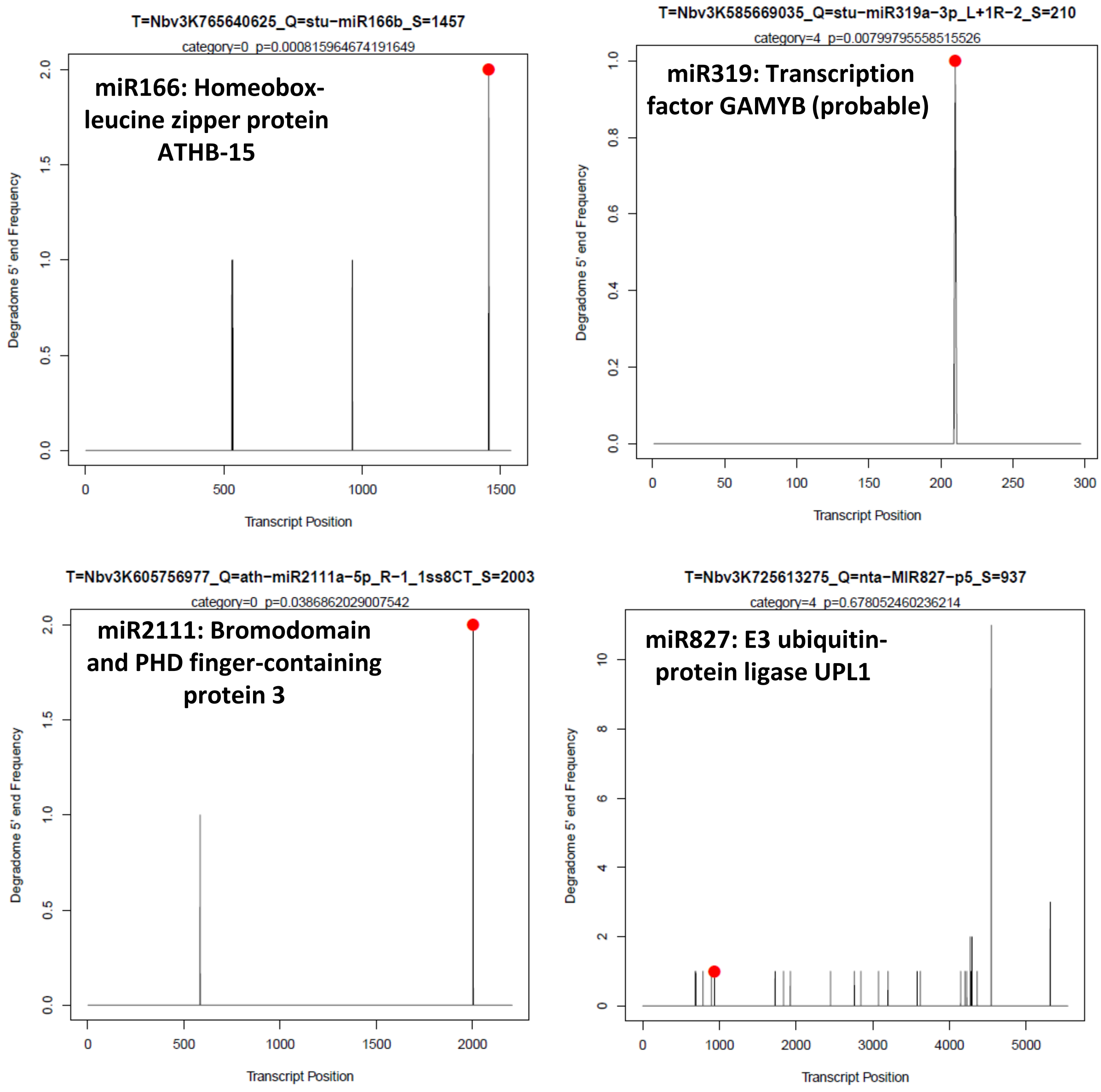

Figure S2 Degradome T-plots for potential miRNA targets. See Figures 6–8 in the main text for 5’-RACE cleavage points of target transcripts.

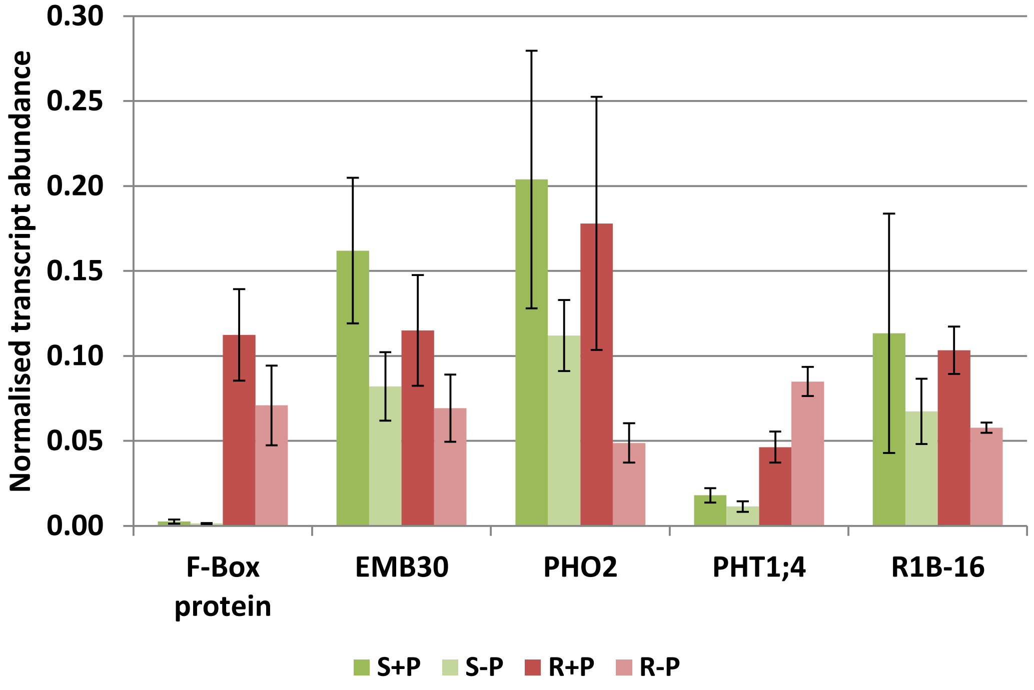

Figure S3 Transcript abundance of targets tested by RLM 5' RACE for cleavage by phosphate starvation-responsive miRNAs. Two-week old *N. benthamiana* plants were grown on phosphate-sufficient (+P) or phosphate-deficient (-P) medium for one week. Shoots (S) and roots (R) were harvested. Average of target transcript abundance was calculated for four independent samples as measured by RT-qPCR using the Roche Lightcycler 480 platform. Error bars indicate ±SE.
